# Is what is beautiful good and still more accurately understood? A replication and extension of Lorenzo et al. (2010)

**DOI:** 10.1177/08902070221099688

**Published:** 2022-05-17

**Authors:** Hasagani Tissera, John E Lydon, Lauren J Human

**Affiliations:** Department of Psychology, 5620 McGill University, Montreal, QC, Canada

**Keywords:** attractiveness, first impressions, accuracy, pre-registered replication, attention

## Abstract

Is what is beautiful good and more accurately understood? Lorenzo et al. (2010) explored this question and found that more attractive targets (as per consensus) were judged more positively and accurately. Perceivers’ specific (idiosyncratic) ratings of targets’ attractiveness were also related to more positive and accurate impressions, but the latter was only true for highly consensually attractive targets. With a larger sample (*N* = 547), employing a round-robin study design, we aimed to replicate and extend these findings by (1) using a more reliable accuracy criterion, (2) using a direct measure of positive personality impressions, and (3) exploring attention as a potential mechanism of these links. We found that targets’ consensual attractiveness was not significantly related to the positivity or the accuracy of impressions. Replicating the original findings, idiosyncratic attractiveness was related to more positive impressions. The association between idiosyncratic attractiveness and accuracy was again dependent on consensual attractiveness, but here, idiosyncratic attractiveness was associated with lower accuracy for less consensually attractive targets. Perceivers’ attention helped explain these associations. These results partially replicate the original findings while also providing new insight: What is beautiful to the beholder is good but is less accurately understood if the target is consensually less attractive.

One of the more robust, highly cited findings in psychology is that attractive people are perceived more positively, which is termed the “what is beautiful is good effect” (Dion et al., 1972). Attractive people are judged and treated more positively than less attractive people, and this effect has been observed across varying domains (e.g., personality, intelligence, health, popularity, social skills), levels of acquaintanceship, cultures, and ages (for meta-analyses, see Eagly et al., 1991; Feingold, 1992; Langlois et al., 2000), resulting in a broad range of implications. Although past work on the attractiveness stereotype has focused on the positivity of impressions, the association between attractiveness and the accuracy of impressions has been left largely unexplored, with the exception of one study (Lorenzo et al., 2010). Given that impressions can simultaneously be characterized by both positivity and accuracy, which are considered independent components from each other (see Fletcher & Kerr, 2010; Gagné & Lydon, 2004), it is important to consider how attractiveness may also relate to the accuracy of impressions. As such, in the present research, we aimed to provide a direct replication and extension of Lorenzo et al.’s (2010) study.

Why is the association between attractiveness and the accuracy of impressions important? Previous research demonstrates that positive and accurate impressions may independently and differentially relate to interpersonal correlates (e.g., Human et al., 2020; Kerr et al., 2020; Neff & Karney, 2005). For example, above and beyond the positivity of personality impressions, there is evidence that forming more accurate impressions in platonic, getting acquainted interactions is related to greater concurrent liking (Human & Biesanz, 2011), and that greater initial accuracy is predictive of greater liking over time (Human et al., 2013, 2020). Thus, given that accuracy at first impressions can have important consequences for relationship initiation, it is important to understand the predictors of accurate impressions, such as the role of attractiveness, the focus of the current research.

## Indexing accuracy

To index accuracy, perceivers’ impressions must be compared to a predetermined benchmark, also referred to as a validity or accuracy criterion, that provide a realistic indicator of a target’s actual personality (Funder, 1995). Based on previous work, possible accuracy criteria include, but are not limited to, the target’s self-reported personality, third-party ratings of the target’s personality (e.g., close-other ratings, objective coder ratings), behavioral coding of the target’s personality (e.g., Connelly & Ones, 2010; Kolar et al., 1996; Oh et al., 2011; Thielmann et al., 2017; Vazire & Mehl, 2008). The gold standard is considered to be some combination of multiple accuracy criteria (Funder, 1995). In the present study, we utilize both self-reports (to parallel the original study) and a combination of self- and close-other reports (to reflect the gold standard) as our accuracy criterion. Furthermore, the round-robin study design employed in this study considers each participant as both a perceiver and a target. Specifically, each participant interacts with every other participant in a given study session, both rating and being rated by their interaction partner, thus allowing each participant to serve as both a perceiver and target across multiple, naturalistic interactions. This allows for more reliable estimates of accuracy, as they are based upon multiple interactions.

There are, however, several different ways to compare perceivers’ impressions to the accuracy criterion, including absolute (e.g., difference score) or more relative (e.g., correlational) approaches. Given the interpretational issues with absolute approaches and difference scores more generally (e.g., Cronbach, 1955; Furr, 2011), we take a relative approach, which can be further characterized as either trait-based or profile-based (see Back & Nestler, 2016 for an in-depth discussion). The trait-based approach is concerned with whether perceivers can correctly judge targets’ standing on a given trait relative to other targets. For example, Paige, the perceiver, must correctly determine Tyler, the target’s, standing on various traits, such as extraversion or conscientiousness, relative to other people. When the judgment of numerous traits is involved, each of these traits must be examined separately.

The profile-based approach (Biesanz, 2010; Cronbach, 1955; Furr, 2008), which we employ in the current article, is concerned with perceivers’ ability to accurately detect targets’ specific patterning on a number of different personality items. For example, Paige, the perceiver, must correctly determine that Tyler, the target, is more friendly than careless. Importantly, the profile-based approach, although technically a person-centered approach, can also provide an indicator of average trait-based accuracy, on average across all the personality items included in the analysis (when profile agreement is decomposed into distinctive accuracy—see below). Thus, profile agreement can also indicate whether Paige is able to distinguish Tyler from other targets on average across all the items in the personality profile (see Biesanz, 2021).

We utilized the profile-based approach—specifically, the componential profile approach using the Social Accuracy Model (SAM; Biesanz, 2010, 2021) – for several reasons. First, this was the approach taken in the original study by Lorenzo et al. (2010), thereby enabling direct comparison to the original results. Second, this approach allows for the examination of multiple meaningful components of profile agreement, including distinctive accuracy, normative accuracy, and positivity, discussed below, each of which may have implications for relationship development in early acquaintanceship (Human et al., 2013, 2020). Third, using SAM, a multilevel modeling approach, to assess each component of profile agreement provides several statistical advantages over the trait-based approach. Specifically, SAM allows for the assessment of each component of accuracy and moderators simultaneously within the same model, with the item being the unit of analysis, thereby retaining all the data in a single model, which maximizes power and minimizes the probability of Type II errors. Furthermore, considering the whole personality profile at once, as opposed to running separate analyses for each trait, reduces the total number of analyses, which helps to minimize the probability of Type I errors. Further, as noted above, this approach can also provide an estimate of the average trait-based accuracy. Moreover, although not the case for the present manuscript, if trait-specific hypotheses or research questions do exist, these can be integrated into the profile-based approach (e.g., Huelsnitz et al., 2020; Liu & Ilmarinen, 2020).

### Normative and distinctive accuracy

Relying on overall profile agreement between perceiver ratings of a target and targets’ accuracy criterion to assess accuracy could artificially inflate accuracy levels. This is because the perceiver could have formed an impression of the target by simply relying on their existing knowledge about people’s typical personality profiles. Given that targets, in general, tend to resemble the personality profile of an average individual, perceivers’ reliance on their existing knowledge could lead to accurate impressions. However, this accuracy level does not necessarily capture the perceiver’s actual knowledge about a specific target. Did Paige the perceiver form an accurate impression of Tyler, the target, because she relied on her knowledge about the normative personality profile, or did she form an accurate impression of Tyler specifically, because of cues he emitted, and she utilized? It is, therefore, important to parse out the different sources of accuracy. One benefit of the profile approach is the ability to decompose accuracy into multiple components. Specifically, using the componential profile agreement approach, we are able to partial out the average person from the link between the targets’ validity measure and the perceivers’ impressions.

As such, the present work considered two types of accuracy: normative and distinctive accuracy (see Table 1 for a summary of all key terms explored in the present research).. Normative accuracy indexes the extent to which a perceiver views a target as being similar to the average person’s personality profile, such as more creative than careless and more outgoing than creative. Put differently, normative accuracy is the extent to which the perceiver, Paige, correctly perceives how the target, Tyler, is similar to the normative personality profile. The normative profile has been found to be socially desirable in general, and therefore it is also considered an indicator of the positivity of impressions (Borkenau & Leising, 2016; Rogers & Biesanz, 2015; Wood & Furr, 2016). That is, the normative and the socially desirable personality profiles are highly correlated, because, in general, people report more positive than negative personality traits.

**Table 1.** Summary of key terms explored in the present study.

Term	Definition
Distinctive accuracy	The extent to which a perceiver accurately views the target’s unique personality profile.
Normative accuracy	The extent to which a perceiver accurately views how the target’s personality profile corresponds to the average person’s (i.e., normative) profile.
Positivity	The extent to which a perceiver accurately perceives how the target’s personality profile is similar to the socially desirable profile.
Consensual attractiveness	A target’s general attractiveness, as indexed by averaging the attractiveness ratings of the target across multiple perceivers.
Idiosyncratic attractiveness	A perceiver’s unique attractiveness rating of a target, adjusted for that target’s consensual attractiveness.
Consensual attention	A target’s general tendency to capture perceivers’ attention, indexed by averaging attention ratings across multiple perceivers.
Idiosyncratic attention	A perceiver’s unique rating of attention for a given target, adjusted for that target’s consensual attention.

As alluded to earlier, there could be different sources of normative accuracy. For example, some perceivers may simply rely on their pre-existing knowledge of base-rate information about personality traits (e.g., people are typically more outgoing than careless), consciously or not, to achieve normative accuracy. In contrast, others may achieve normative accuracy by paying attention to and correctly understanding how normative a specific target actually is. Thus, it is not clear to what extent normative accuracy is due to chance, as opposed to truly indexing a perceiver’s actual knowledge about a specific target.

Complementing normative accuracy is the second component, distinctive accuracy, which indexes the extent to which a perceiver recognizes the unique patterning of a target’s personality profile, that is different from the normative profile. In other words, this is the extent to which Paige is able to correctly identify Tyler’s specific patterning of personality traits (e.g., more shy than reliable, relative to most people). Although normative accuracy may be considered an index of positivity, distinctive accuracy is considered neutral in terms of valence, because people in general can deviate from the normative in even more positive ways or in more negative ways.

The normative and the distinctive components of accuracy are independent of each other (e.g., Fletcher & Kerr, 2010; Funder & Colvin, 1997; Gagné & Lydon, 2004; Kerr et al., 2020; Leising et al., 2016), and therefore may be related to correlates in different ways. Although intuitively it may seem difficult to judge the distinctive profiles of those targets who are more “normative,” this is not necessarily the case. In line with this reasoning, previous work has demonstrated that people higher in well-being (who tend to possess more normative personality profiles; Wood et al., 2007) were seen with greater distinctive accuracy (e.g., Human et al., 2014; Human et al., 2019).

Furthermore, in contrast to normative accuracy, distinctive accuracy is a clearer indicator of the perceiver’s actual knowledge about a target, as it is more difficult to achieve distinctive accuracy by chance. As a result, people generally display relatively lower levels of distinctive accuracy than normative accuracy (e.g., Biesanz et al., 2011; Human & Biesanz, 2011; Human et al., 2014; Krzyzaniak et al., 2019; Lorenzo et al., 2010; Rauthmann & Sherman, 2017), as it requires greater resources, such as attention and cognitive effort. Given the different underlying processes relating to normative and distinctive accuracy, and that they are independently associated with interpersonal consequences such as liking (e.g., Human et al., 2020), there is value in exploring these two components separately to better understand how predictors, such as target’s attractiveness, might (or might not) differentially relate to distinctive and normative accuracy.

### Links between attractiveness and normative and distinctive accuracy of first impressions

The focus of the present replication study is to further the understanding of how attractiveness might relate to normative and distinctive accuracy of personality impressions during first impressions. Thus far, only one study has examined the association between targets’ attractiveness and normative and distinctive personality accuracy. In a getting-acquainted round-robin study in which multiple perceivers met multiple targets face-to-face for several minutes (Lorenzo et al., 2010), two forms of attractiveness were examined: consensual and idiosyncratic. Consensual attractiveness is a target’s mean attractiveness score on average across all of the perceivers they met with. Thus, consensual attractiveness reflects the target effect of attractiveness. For example, if Tyler met with Paul, Paige, Piper, and Phoebe, who rated Tyler’s attractiveness as 6, 7, 6, and 5, respectively, then Tyler’s consensual attractiveness score would be 6. In other words, this is how attractive a target is viewed to be in general, across perceivers.

Idiosyncratic attractiveness is how attractive a specific perceiver found that target, above and beyond the target’s consensual attractiveness level, reflecting a dyadic effect of attractiveness. This is computed by subtracting the target’s average attractiveness score from the perceiver’s raw attractiveness rating of the target. For example, Paige’s idiosyncratic rating of Tyler would be 1 because her unique rating of Tyler, which was 7, was one point above Tyler’s consensual attractiveness score, which was 6. Thus, idiosyncratic attractiveness is a perceiver’s unique rating of a target’s attractiveness, adjusted for that target’s consensual attractiveness level.

How were these indices associated with accuracy? Lorenzo et al. (2010) found that targets who were higher in consensual attractiveness were perceived more in line with their unique self-reported profile of personality traits (i.e., distinctive accuracy), and more in line with the typical, socially desirable personality profile (i.e., normative accuracy). In other words, targets who were judged as being highly attractive by a group of people were seen with greater distinctive and normative accuracy. In addition, perceivers’ idiosyncratic attractiveness ratings were related to greater normative accuracy. That is, when a perceiver found a target to be especially attractive, even more so than most other perceivers, they viewed that target as more similar to the normative, socially desirable personality profile. Idiosyncratic attractiveness was also related to distinctive accuracy, but the association was conditional on targets’ consensual attractiveness. Specifically, viewing a target as especially attractive, compared to other perceivers, was associated with greater distinctive accuracy, but only for targets who were consensually rated as being highly attractive. For example, if Paige rated Tyler, a consensually attractive target, as highly attractive, then she is likely to perceive Tyler’s unique personality more accurately. Thus, based on these findings, attractiveness, both more objective (i.e., consensual) and more subjective (i.e., idiosyncratic), may promote both distinctive and normative accuracy (and positivity) of personality impressions. Given the limited work on this topic, the present study provides a replication and extension of these associations using a larger sample to test the reliability of these associations.

### Why might attractiveness relate to normative accuracy?

Attractiveness could relate to normative accuracy in several ways. Consistent with research on the attractiveness stereotype (Eagly et al., 1991; Feingold, 1992; Langlois et al., 2000), perceivers may attribute more positive, socially desirable personality profiles to more attractive targets. This could contribute to greater normative accuracy as some evidence suggests that more consensually attractive targets may possess relatively more desirable traits such as agreeableness and extraversion (Meier et al., 2010), although the evidence is mixed (see Feingold, 1992). If so, perceivers could simply rely on their normative knowledge, which tends to be positive in general, to infer the personalities of these targets, in turn enhancing normative accuracy. Another mechanism underlying the link between attractiveness and normative accuracy could be attention. More attractive targets may capture perceivers’ attention to a greater extent and perceivers’ idiosyncratic attractiveness ratings of targets could also promote perceivers’ attention towards these targets. With greater attention, perceivers could better detect the extent to which a target is actually similar to the normative personality profile. As such, both consensual and idiosyncratic attractiveness could be related to greater normative and positive personality impressions.

### Why might attractiveness relate to distinctive accuracy?

According to Funder’s Realistic Accuracy Model (*RAM:* Funder, 1995), accurate impressions require successfully navigating four steps, in which targets make (1) *relevant* cues of their personalities (2) *available* to the perceiver, who must (3) *detect* and correctly (4) *utilize* these cues in forming judgments. Are there specific characteristics of certain targets that could facilitate this process? One characteristic that may play a role is social confidence, as people who are more comfortable socially and with themselves may be more likely to express relevant cues to their personalities. This could be an active process motivated by a desire to be seen in line with their self-views as predicted by self-verification theory (Swann, 1983), or a more unintentional result of social ease. Given that past work has found support that more consensually attractive targets tend to be more socially confident (Eagly et al., 1991; Feingold, 1992; Reis et al., 1980), this could enable them to express more relevant cues about their personalities, which could enhance distinctive accuracy. Consistent with this idea, previous work has found that well-adjusted individuals, including those high in self-esteem, are more likely to provide more relevant cues of their personality, which contributes to being seen with greater distinctive accuracy (Human et al., 2014, 2019). Conversely, targets who are less attractive may be less socially confident, which could lead them to provide less relevant cues about their personalities, hindering distinctive accuracy.

Once targets have conveyed relevant cues about their personalities, perceivers must correctly detect and utilize these cues to form judgments about the targets. What might facilitate this process? Perceivers’ idiosyncratic perceptions of targets’ attractiveness may promote attention (Langlois et al., 2000; Maner et al., 2003), which could help perceivers better detect cues that may act as indicators of targets’ personalities, in turn enhancing normative and distinctive accuracy (Capozzi et al., 2019; Human et al., 2012). But this may only be true for more consensually attractive targets if less consensually attractive targets do not convey as relevant cues. For example, Paige’s attention toward Tyler may only contribute to greater accuracy to the extent that Tyler conveys relevant cues about his personality to Paige, which may require a certain level of social confidence. This is in line with the multiplicative nature of the RAM, which suggests that the later stages (e.g., detection) will only facilitate accuracy if earlier stages (e.g., relevance) are achieved. Thus, there is reason to believe that both targets’ consensual attractiveness and perceivers’ idiosyncratic attractiveness ratings of targets may promote the accuracy of first impressions, although the latter association may depend on the targets’ consensual attractiveness level.

### Beyond direct replication

The present research goes beyond directly replicating Lorenzo et al. (2010)’s findings to extend them in several respects. First, the present research is further built on the original study by using a composite of self- and close-other ratings of targets’ personalities, a more reliable accuracy criterion than self-reports only (Kolar et al., 1996). Second, the present study also extended the original study (Lorenzo et al., 2010) by considering the positivity of impressions separately from normative accuracy. We expand on each of these points below. Third, we explored the possible role of attention in the associations between attractiveness and the accuracy of first impressions. We expand on each of these points below.

### Reliability of the accuracy criterion

Previous work has found that close-other reports are not redundant with self-reported personality and have unique predictive validity of a target’s personality, above and beyond a target’s own self-reported personality (Vazire, 2010; for a meta-analysis, see; Connelly & Ones, 2010). Further, Connelly and Ones (2010) have found that combining self- and close-other reports is a stronger predictor of a target’s behavior at first impressions, than when considering a target’s self-ratings or close-others' reports on their own. As such, in a second set of analyses, we utilized a more reliable accuracy criterion, the average of self- and close-other reports of targets’ personality, to see whether the links between attractiveness and accuracy are persistent.

### Parsing out positivity and normative accuracy

The original study relied on normative accuracy as an indicator of the positivity of impressions. Even though the normative and the socially desirable personality profiles are highly correlated, recent work has demonstrated that it is possible and recommended to distinguish them when positivity is of primary interest (Rogers & Biesanz, 2015; Wessels et al., 2020; Zimmermann et al., 2018).^1^ This is because one may achieve normative accuracy by either relying on their knowledge of the average person’s profile, or by simply attributing a more positive personality profile to the target. Therefore, to better understand what is driving the link between attractiveness and normative accuracy observed in Lorenzo et al.’s (2010) work, it is important to disentangle normative (i.e., neutral) knowledge from the positivity of impressions. As such, in the present study, in a separate set of analyses, we distinguished positivity from normative accuracy using more recent guidelines for extending the model used in the original study.

### Considering attention as a mechanism

As explained above, attention is a likely mechanism that may underlie the associations between attractiveness and normative and distinctive accuracy. That is, greater attention could allow perceivers to better detect the target’s cues regarding their normative and more distinctive personality profiles. In this way, attention could contribute to both greater levels of normative and distinctive accuracy. In the present research, we parse out the dyadic versus target effects of attention to better understand the source of attention that is driving these links. Thus, as with attractiveness, the present study considered two indices of attention: consensual and idiosyncratic. Consensual attention captures the target effect of attention. That is, to what extent is the target (e.g., Tyler) interesting and captivating, on average, across multiple interactions? Therefore, consensual attractiveness is the extent to which a target is generally attention-getting. This is computed by averaging the attention scores given to a target by multiple perceivers. If Paul, Paige, Piper, and Phoebe give scores of 3, 4, 6, and 7 in terms of how much they paid attention to Tyler, then Tyler’s consensual attention score will be a 5.

Idiosyncratic attention captures the dyadic effect. That is, to what extent does Paige, the perceiver, rate Tyler as interesting, above and beyond how interesting Tyler typically tends to be? In other words, idiosyncratic attention represents the unique attention ratings given by a perceiver to a target, adjusted for that target’s consensual attention. For instance, Paige’s idiosyncratic attention score is −1 as she gave an attention score of 4 to Tyler, which was a point below Tyler’s consensual attention score. It is important to separately examine these components because otherwise, raw attention ratings can be conceptually difficult to interpret. For example, if Paige reports a score of 4 for how engaging and interesting Tyler was, then it is not clear whether this is because of Tyler’s general attention-getting tendencies or because Paige found Tyler more captivating, even more than his usual tendencies. As such, the present research examined consensual and idiosyncratic attention as potential contributors to the links between attractiveness and accuracy. This distinction permitted for a more in-depth interpretation of the results as it allowed us to attribute the source of attention to either the target or the dyad.

## Summary of present study aims

The purpose of the current pre-registered study was three-fold. First, we conducted a direct replication of Lorenzo and colleagues’ (2010) original study to determine the robustness of the original pattern of results. Specifically, we used the same round-robin design and modeling approach on a much larger sample with similar characteristics as in the original study. Second, we extended the original model by (a) using a more reliable accuracy criterion, the average of both target and close-other reports of a targets’ personality, and (b) parsing out positivity from normative accuracy, to determine whether a similar pattern of results is found with these more current, state-of-the-art approaches. Third, we explored whether perceivers’ attention could contribute to the associations between attractiveness and the accuracy of impressions, in an effort to shed initial light on why attractiveness may influence accuracy.

## Method

The present study was conducted using an existing dataset, and therefore, the study design was not pre-registered. However, we pre-registered all analyses related to the present manuscript on Open Science Framework on the 1st of May, 2019 (https://osf.io/e8b5d). The analytical approach appearing in the pre-registration also appears here, with any deviations clearly marked as such. The study codebook and R code and data for replicating all pre-registered analyses can be found on the Open Science Framework (https://osf.io/64txm/).^2^

### Sample

All study procedures were approved by the university’s institutional research ethics board. Participants were recruited through flyers posted around campus, and in the community (e.g., grocery stores, cafés), as well as through the psychology participant paid and extra credit pools. The sample size in the original study was 73 participants (56 females, 17 males, *M*_*age*_ = 19.38 years, *SD* = 1.60), consisting of 252 dyadic interactions. The current sample consists of 547 undergraduate students (80 males, 464 females, 3 did not disclose; *M*_*age*_ = 20.42 years, *SD* = 2.14) at a different institution, recruited across two waves of data collected between the years 2016–2017 and 2017–2018. Dyads who previously knew each other (*n = *204) were excluded from analyses. Two participants did not provide self-reported personality ratings and therefore, were not considered as targets in the present analyses (*N*_targets_ *=* 545). One participant did not provide ratings of their interaction partners, and therefore, they were not considered as a perceiver in the present analyses (*N*_perceivers_ *=* 546). Following these exclusions, the final sample consisted of 2851 dyads. Participants were compensated 2 course credits or $20.00 for their participation.

### Statistical power

We examined whether there was sufficient statistical power for the present analyses. To this effect, we conducted two types of power analyses to approximate achieved power in the present sample. First, we used the fabs package for R (github\jbiesanz\fabs) to compute the expected power for detecting the observed associations between (distinctive or normative) accuracy and (consensual or idiosyncratic) attractiveness in Lorenzo et al. (2010). This approach uses a Bayesian framework to address the issues of effect size heterogeneity and sampling error (see Biesanz & Schrager, 2017 and McShane & Böckenholt, 2016 for further discussion on expected power). As such, by examining the posterior distribution of an initial effect size parameter, this method of estimating power formally takes into account the uncertainty of an observed effect size. To address the issue of publication bias and the probability of null results being filtered, we specified a filter value of 90%, based on previous work on the publication bias (Ingre & Nilsonne, 2018).

Using this approach, we calculated the power for each of the primary observed effects in Lorenzo et al. (2010). Because the authors did not provide effect sizes in the original manuscript, we converted t-values provided in the paper to Pearson’s *r* correlations using the formula provided by Rosenthal and Rosnow (2007): $r=\;\sqrt{\frac{t^{2}}{t^{2}+df}}$ . Of note, as the present research involves multilevel models, effect sizes are approximate. Given the original sample size (*N* = 73) and the observed effect sizes for the links between (a) consensual attractive and normative accuracy (*r* = 0.47), (b) consensual attractive and distinctive accuracy (*r* = 0.32), and (c) idiosyncratic attractiveness and normative accuracy (*r* = 0.56), we concluded that the present sample of N_perceivers_ = 546 provides between 90% and 99% power to detect each of these associations.

Second, we also attempted to determine whether there was sufficient power to detect the interaction effect observed in Lorenzo et al. (2010) between distinctive accuracy, consensual attractiveness, and idiosyncratic attractiveness. Although this is examined as a three-way interaction using profile analysis in the present work, it is conceptualized as a two-way interaction effect (i.e., does consensual attractiveness moderates the link between idiosyncratic attractiveness and distinctive accuracy?). Indeed, we ensured that the pattern of results obtained with the profile approach was the same with the saved-out distinctive accuracy scores. To do so, we extracted the empirical Bayes estimates for the dyadic random effects, and examined whether idiosyncratic and consensual attractiveness interacted to predict distinctive accuracy, which provided the same results as the SAM analyses. We then computed the expected power for a moderation effect. The approach used to estimate power for the two-way associations was not able to estimate power for interaction effects, and therefore, we used a different statistical package to estimate power here. Using the InteractionPoweR (Baranger, 2021) package for *R,* we calculated the power achieved for testing a two-way interaction. Effect size estimates (*r*) used for this analysis were computed by converting the observed t-values from Lorenzo et al. (2010), as described above. Specifically, power was based on the interaction effect between consensual and idiosyncratic attractiveness to predict distinctive accuracy (*r =* .28), the link between consensual attractiveness and distinctive accuracy (*r* = .32), the link between idiosyncratic attractiveness and distinctive accuracy (*r =* 0), and the link between consensual and idiosyncratic attractiveness (*r* = 0). Specific steps for deriving these estimates are detailed in the R script. Based on 1000 simulations, results revealed that the present data provided over 99% power to detect this effect. Overall, these analyses suggest that the present study provided sufficient power to detect all the primary effects observed in Lorenzo et al. (2010), though we acknowledge again that the effect size estimates and approaches are all approximations. The R code for the analyses with the saved-out distinctive accuracy scores and the power analyses is available on Open Science Framework: https://osf.io/64txm/.

Finally, we note that there are different ways to estimate power for multilevel models. One popular approach is to conduct simulation studies. Although we did not conduct simulations for estimating power, considering the rules of thumb derived from simulations may shed further light on whether we had sufficient power in the current sample. For example, Arend and Schäfer (2019) indicate that, as a rule of thumb, one can achieve 80% power to detect a medium cross-level interaction effect size with a medium random slope, and with *N* = 150 as the level 2 sample size and *n* = 16 as the Level 1 sample size. The present sample far exceeds these requirements as we conducted profile analyses employing 21 items, 545 targets, and 2851 dyadic interactions, which result in a total of 68,401 observations in the dataset.

### Procedure

In the original study, participants arrived at the lab in groups of 5 to 11 people (*Mdn* = 7). Participants engaged in a round-robin paradigm, where they met one-on-one with every other participant present at the session for 3 minutes. Following each brief meeting, participants reported on their interaction partner’s personality traits and physical attractiveness. Participants’ self-ratings of their own personality profile were also collected using the same items. The present study procedures closely followed those reported in Lorenzo et al. (2010). Specifically, in the present study, participants arrived at the lab in groups ranging in size from 4 to 8 people *(Mdn = 8*). First, participants completed an initial survey in which they reported on their personality. They also provided contact information of three close-others (e.g., friends, family members, romantic partner), who were later contacted on behalf of the participants to complete personality ratings about the participant. Next, participants engaged in 2–3 minute, one-on-one getting acquainted interactions with every other participant present at the session. Following each meeting, participants reported on their impressions of the target’s personality traits, the extent to which they found the target physically attractive, and the extent to which they were engaged in the interaction (the indicator of attention). Finally, participants completed more individual difference measures that were not examined for this manuscript.

### Measures

#### Personality ratings

Participants and close others provided personality ratings using the full 44-item version of the Big Five Inventory (John & Srivastava, 1999). As in the original study, three items were included to measure intelligence: “is intelligent,” “is bright,” and “receives good grades.” During the round-robin, each interaction partner’s personality was rated using the same 21-item version of the BFI, with the addition of the three items for measuring intelligence, as in the original study (Lorenzo et al., 2010). The subset of 24 items that overlapped for perceivers, targets, and targets’ close others was used for the personality impression analyses (see the supplementary online materials (SOM) for self- and other-correlations for each of the personality items). All items for self-ratings and partner-ratings were rated on a 7-point Likert scale ranging from (1) *disagree strongly* to (7) *agree strongly*, which is identical to the response options provided in the original study.

#### Physical attractiveness

Following each interaction, participants also rated their interaction partner’s physical attractiveness. In the original study, participants reported on “how physically attractive” the other person was on a scale of (1) *not at all* to (7) *a great deal.* In the present study, participants were asked to rate the item “I see this person as someone who is physically attractive” (*M* = 5.03, *SD* = 1.21). This item was rated on a Likert scale ranging from (1) *disagree strongly* to (7) *agree strongly.* Perceiver’s idiosyncratic perceptions of attractiveness were parsed out from targets’ consensual attractiveness level in the same way as it was in the original paper. As in the original study, an index of consensual attractiveness for each target was created by saving out the empirical Bayes estimates of the average of all perceivers’ ratings of attractiveness for that same target. This approach is analogous to computing the average attractiveness rating for each target across several perceivers. Targets’ consensual attractiveness scores were then subtracted from perceivers’ individual dyadic ratings to index perceivers’ idiosyncratic attractiveness, adjusted for the targets’ consensual attractiveness.

#### Attention

Participants’ engagement with the target was used as a proxy for their attention. Specifically, participants rated the item “This participant was engaging and interesting” about their interaction partner on a 7-point Likert scale ranging from (1) *disagree strongly* to (7) *agree strongly* (*M* = 5.46, *SD* = 1.03). To parallel the analyses with physical attractiveness and to parse out perceivers’ idiosyncratic attention and the targets’ general tendency to be attention-getting, an index of consensual attention was created for each target by saving out the empirical Bayes estimates of the average target engagement across all perceivers, which was then subtracted from perceivers’ dyadic attention ratings. Although we pre-registered analyses with attention, the use of both consensual and idiosyncratic attention was not pre-registered. However, this was simply due to oversight, as it is important and more conservative to have both predictors in the model to parse out perceiver effects from target effects, and parallels the approach with attractiveness, which was pre-registered.

### Analytical approach

All analyses were conducted using *R’s lme4* package (Bates et al., 2015). We pre-registered running four models using the Social Accuracy Model (*SAM:* Biesanz, 2010) procedures: (1) a direct replication of the original study with distinctive and normative accuracy, using target self-reports as the accuracy criterion, (2) an extension of the original model using a more reliable accuracy criterion: the average of self- and close-other reports, (3) an extension of the original model using an extended model parsing out impressions into distinctive accuracy, normative accuracy and positivity but retaining self-reports as an accuracy criterion, and (4) the fully extended model with the more reliable accuracy criterion (the average of self- and close-other reports). For simplicity, we only report the results of models 1 (Direct Replication Model) and 4 (Fully Extended Model) here and provide the results for models 2 and 3 in the SOM and discuss them briefly in the discussion section.^3^

#### Direct replication model

As in the original study, we conducted multilevel analyses following the guidelines from the Social Accuracy Model (Biesanz, 2010). Specifically, we predicted grand-mean centered perceiver ratings of targets’ personality from (1) the normative personality profile, which was computed by taking the sample means for each of the personality items, and (2) the accuracy criterion for targets’ personality, which, in this model, was targets’ distinctive self-ratings of each of the personality items. Targets’ distinctive ratings were computed by subtracting out the normative means from targets’ self-reported personality. This step minimizes model convergence issues and enhances the interpretability of constructs (for further discussion see Biesanz, 2010; 2021). As per the guidelines outlined by the SAM (Biesanz, 2010) and consistent with Lorenzo et al. (2010), both predictors (the normative profile and the distinctive target’s self-rated profile) were also grand-mean centered prior to analyses and individual personality items were not reverse coded in the analyses to maintain the spread of the personality traits in the profile. This model is outlined in equation (1).

(1) $$Y_{pti}=\;\beta_{0pt}+\beta_{1pt}{\mathrm{NormativeMeans}}_{i}+\;\beta_{2pt}{\mathrm{DistinctiveTargetRatings}}_{ti}+\;e_{pti}$$

In the above equation, grand-mean centered perceiver ratings are represented by $Y_{pti}$ , indicating perceiver *p’s* ratings of target *t* on personality item *i.* ${\mathrm{NormativeMeans}}_{i}$ represents the grand-mean centered sample means of the average targets’ self-reported ratings for each personality item *i*. As such, $\beta_{1pt}$ is the extent to which perceiver *p*’s ratings of target *t*’s personality on item *i* correspond to the averaged target’s self-reports for item *i.* This is considered the normative accuracy slope—the extent to which perceivers see the target as having a normative, and therefore socially desirable, personality profile.

Next, ${\mathrm{DistinctiveTargetRatings}}_{ti}$ represents our accuracy criterion for item *i*, target *t’*s distinctive self-reported personality, which has been centered within item (i.e., the normative means were subtracted from target ratings). As such, $\beta_{2pt}$ represents the extent to which perceiver *p*’s ratings of target *t* on item *i* correspond to target *t*’s unique self-report on item *i*, with the normative personality profile subtracted out. This is the distinctive accuracy slope—the extent to which perceivers recognize how the target’s self-reported personality deviates from the average person.

Next, we examined the roles of targets’ consensual attractiveness ( ${\mathrm{Consensual}}_{t}$ ) and perceptions of idiosyncratic attractiveness ratings of each target ( ${\mathrm{Idiosyncratic}}_{pt}$ ), adjusted for consensual attractiveness. Both predictors were grand-mean centered prior to analyses and were added as predictors of distinctive and normative accuracy slopes, as described in equation (2), based on the SAM equation (1).

(2) $$\begin{array}{l} \beta_{0pt}=\beta_{00}+\beta_{01}{\mathrm{Consensual}}_{t}+\beta_{02}{\mathrm{Idiosyncratic}}_{pt}+\;u_{0p}+\;u_{0t}+\;u_{0pt}\\ \beta_{1pt}=\beta_{10}+\beta_{11}{\mathrm{Consensual}}_{t}+\beta_{12}{\mathrm{Idiosyncratic}}_{pt}+\;u_{1p}+\;u_{1t}+\;u_{1pt}\\ \beta_{2pt}=\beta_{20}+\beta_{21}{\mathrm{Consensual}}_{t}+\beta_{22}{\mathrm{Idiosyncratic}}_{pt}+u_{2p}+\;u_{2t}+\;u_{2pt} \end{array}$$

In this equation, $\beta_{11}$ indicates whether the linear association between the normative mean ratings for item *i* and perceiver *p’s* ratings of target *t* is moderated by target *t’s* consensual attractiveness. This interaction can be conceptualized as a main effect of consensual attractiveness on normative accuracy (i.e., were more consensually attractive targets viewed with greater normative accuracy?). Similarly, $\beta_{21}$ indicates whether the linear association between target *t’*s accuracy criterion and perceiver *p’s* ratings of target *t* is moderated by target *t’s* consensual attractiveness. This interaction can also be conceptualized as a main effect of consensual attractiveness on the distinctive accuracy of impressions (i.e., were more consensually attractive targets viewed with greater distinctive accuracy?). In a similar way, $\beta_{12}$ and $\beta_{22}$ represent the main effects of idiosyncratic attractiveness on both normative and distinctive accuracy. The intercepts and the slopes were allowed to vary randomly by perceiver $u_{p}$ , target $u_{t}$ , and dyad $u_{pt}$ , when possible. Occasionally, consistent with previous work (e.g., Kerr et al., 2020), some random intercepts were dropped to address convergence issues. We attempted to always model the random slopes as they are conceptually more meaningful and provide information on the perceiver and target variability in the levels of distinctive and normative accuracy (Biesanz, 2021). At times (e.g., in Model 3 and 4), dyadic random effects were not modeled due to convergence issues (see Rogers & Biesanz, 2019). However, results were similar with the dyadic random effects included, indicating that neither the convergence issues nor the inclusion of dyadic random effects had an impact on the results reported here.

#### Fully extended model

To separate normative accuracy and positivity and to examine whether attractiveness was similarly linked to distinctive accuracy when using a composite accuracy criterion, we used the model outlined in equation (3)

(3) $$\begin{array}{l} Y_{pti}=\;\beta_{0pt}+\beta_{1pt}{\mathrm{DesirabilityMeans}}_{i}+\beta_{2pt}{\mathrm{NormativeMeans}}_{i}\\ +\;\beta_{3pt}{\mathrm{DistinctiveCompositeRatings}}_{ti}+e_{pti} \end{array}$$

This model was highly similar to the previous model described in equation (1) with a few changes. First, ${\mathrm{DistinctiveCompositeRatings}}_{ti}$ was computed by taking the average of close-other reports of target *t*’s personality on item *i* and then combining it with target *t’s* self-rated personality on item *i*. Second, there was an additional predictor in the model: ${\mathrm{DesirabilityMeans}}_{i}$ , which refers to the grand-mean centered mean social desirability ratings of item *i,* Social desirability ratings were collected from a different sample (*n* = 30) from the same population, where participants rated the extent to which each personality item is socially desirable on a 7-point Likert scale. This is conceptualized as the positivity of personality ratings. As such, the positivity slope ( $\beta_{1pt}$ ) can be interpreted as the extent to which perceivers viewed the targets’ personality in a socially desirable way. Third, following recommendations (Biesanz, 2021), in addition to subtracting the normative item-means from the accuracy criterion ( ${\mathrm{DistinctCompositeRatings}}_{ti}$ ), we also subtracted the social desirability item-means from the normative profile ( ${\mathrm{NormativeMeans}}_{i}$ ) prior to grand-mean centering the normative item-means. Finally, to examine whether consensual and idiosyncratic attractiveness predict distinctive accuracy, normative accuracy and positivity, again, we included grand-mean centered consensual attractiveness ( ${\mathrm{Consensual}}_{t}$ ) and idiosyncratic attractiveness ( ${\mathrm{Idiosyncratic}}_{pt}$ ) as moderators, similar to equation (2).

**The Role of Attention**. We also examined whether perceivers’ attention accounted for any significant associations with attractiveness and accuracy in the extended model. Both predictors of attention were grand-mean centered prior to analyses. Here, we report on whether idiosyncratic attention helped account for the interaction between consensual and idiosyncratic attractiveness to predict the distinctive accuracy slope. We provide further details on these analyses in the results section. For additional pre-registered analyses with attention, please refer to the SOM.

#### Effect size estimates

Although it was not pre-registered, to provide an estimate of effect size for all primary two-way associations, we computed standard effect sizes (*d*’s), which were calculated as the change in distinctive accuracy, normative accuracy or positivity slopes for an increase in 2 standard deviations in the predictor of interest. More precisely, the standardized effect sizes were computed as follows: *d =* $2*\;\left(b*\;\;SD_{x}/SD_{y}\right)$ *,* where *b* is the regression coefficient of interest, *SDx* is the standard deviation of the predictor of the accuracy slope, and *SDy* is the random effect standard deviation for the accuracy slope of interest. As explained by Gelman (2008), by multiplying the standardized coefficients by 2 lend these scores to be comparable to effect sizes for binary independent variables (e.g., Cohen’s *d’s*)*.* As the current study has a large number of observations, we estimated 95% confidence intervals using the “Wald” method in the *lme4* package. A similar approach has been employed in previous research employing the social accuracy model (e.g., Human et al., 2019; Human et al., 2020; Kerr et al., 2020; Wessels et al., 2020; Zimmermann et al., 2018) allowing for easier comparison with past work.

### Results

#### Direct replication model

In general, participants demonstrated both normative accuracy, *b* ***=*** 0.82, *t* = 40.29, *p* < .001, and distinctive accuracy, *b* ***=*** 0.09, *t* = 9.04, *p* < .001. These slopes indicate that perceivers accurately viewed targets in line with the normative, and the more positive, personality profile, while they also correctly recognized targets’ more unique personality profiles. Although distinctive accuracy levels seemed to be somewhat weaker in the present study than the levels reported in the original study (*b* = 0.18; Lorenzo et al., 2010), these levels are comparable to other work in the context of first impressions that have employed a similar study design and analytical approach (range: *b*s = 0*.*07–0.21; Chan et al., 2011; Human et al., 2019; Human et al., 2020). These results are also in line with the general trend observed in the literature where normative accuracy tends to be much higher than distinctive accuracy (range: *b*s = 0*.*75–0.96; Chan et al., 2011; Human et al., 2019; Human et al., 2020; Lorenzo et al., 2010), given this is a much easier task, especially in first impressions, where understanding what makes a person different from the normative profile would be quite challenging.

##### Attractiveness and normative accuracy

**Figure 1.**
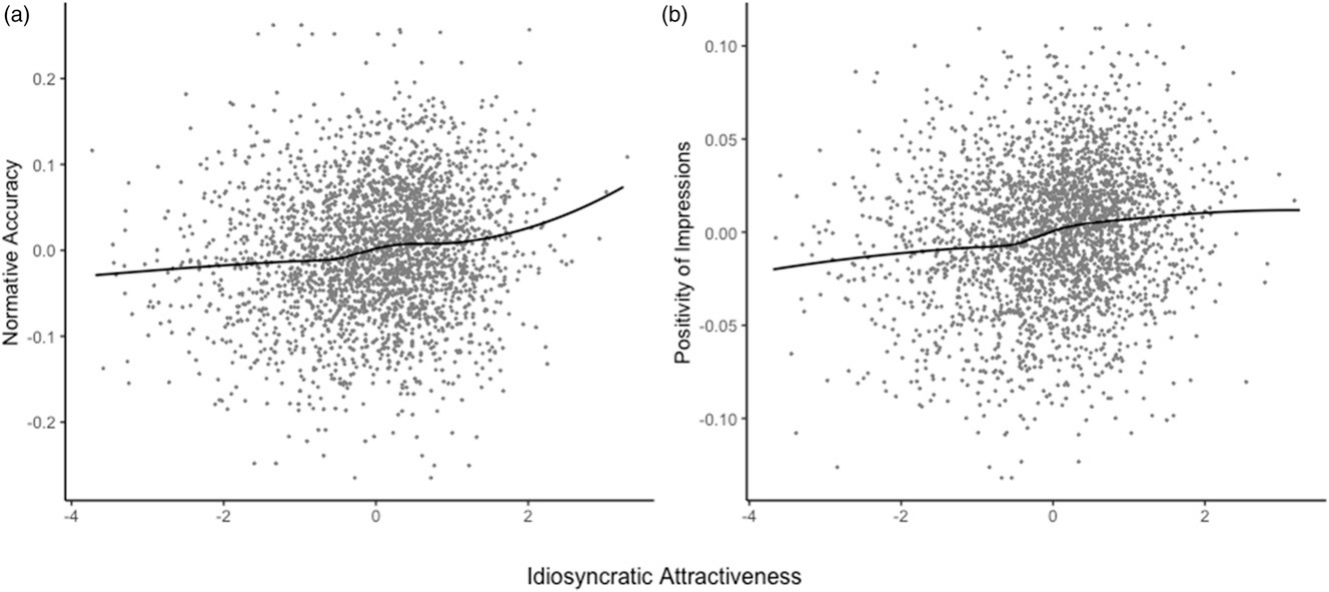
Association between idiosyncratic impressions of targets’ attractiveness and the positivity of personality impressions. *Note.* The nonparametric loess curve in the scatterplot in panel A represents the positive association between idiosyncratic attractiveness and normative accuracy of impressions in the Direct Replication Model. In panel B, the nonparametric loess curve represents the positive association between idiosyncratic attractiveness and positivity of impressions in the Fully Extended Model. All variables were grand-mean centered.

Does targets’ consensual attractiveness relate to normative and distinctive accuracy of personality impressions? Normative accuracy was significantly related to greater consensual attractiveness, *b* = 0.05, *d* = 0.22, *95% CI* [0.05, 0.38]*, t* = 2.51, *p* = .012, and greater idiosyncratic attractiveness, *b =* 0.11, *d* = 0.64, *95% CI* [0.55, 0.74], *t* = 13.26, *p* < .001 (see Figure 1, Panel A), which is consistent with the findings from the original study (consensual attractiveness: *b* = 0.18; idiosyncratic attractiveness: *b* = 0.09; Lorenzo et al., 2010). Thus, targets who were generally viewed as more attractive were more accurately perceived in line with the normative, and more positive, personality profile, in support of the “what is beautiful is good” effect. Further, when perceivers’ viewed a target as especially attractive, they also tended to view them with greater normative accuracy and therefore positivity, supporting that what is beautiful to the beholder is good. Lastly, consensual attractiveness was not a significant moderator of the association between idiosyncratic attractiveness and positivity of impressions, *b* ***=*** 0.01, *t* = 1.03, *p* = .302.

##### Attractiveness and distinctive accuracy

Next, we examined how consensual and idiosyncratic attractiveness were related to distinctive accuracy. Contrary to the original findings (*b = .*09; Lorenzo et al., 2010), consensual attractiveness was not significantly related to greater distinctive accuracy, *b* = 0.002, *d* = 0.08, *95% CI* [−0.88, 1.04]*, t* = 0.17, *p* = .866. Furthermore, unexpectedly, greater idiosyncratic attractiveness was associated with significantly lower distinctive accuracy, *b* = −0.01, *d* = −0.63 *95% CI* [−1.03, −0.22], *t* = −3.00, *p* = .003 (the parallel association was not reported in Lorenzo et al., 2010). This suggests that perceivers were not necessarily more accurate in viewing the unique personalities of targets who were generally viewed as more attractive, and viewing a target as especially attractive was actually associated with viewing their unique personality less accurately.

**Figure 2.**
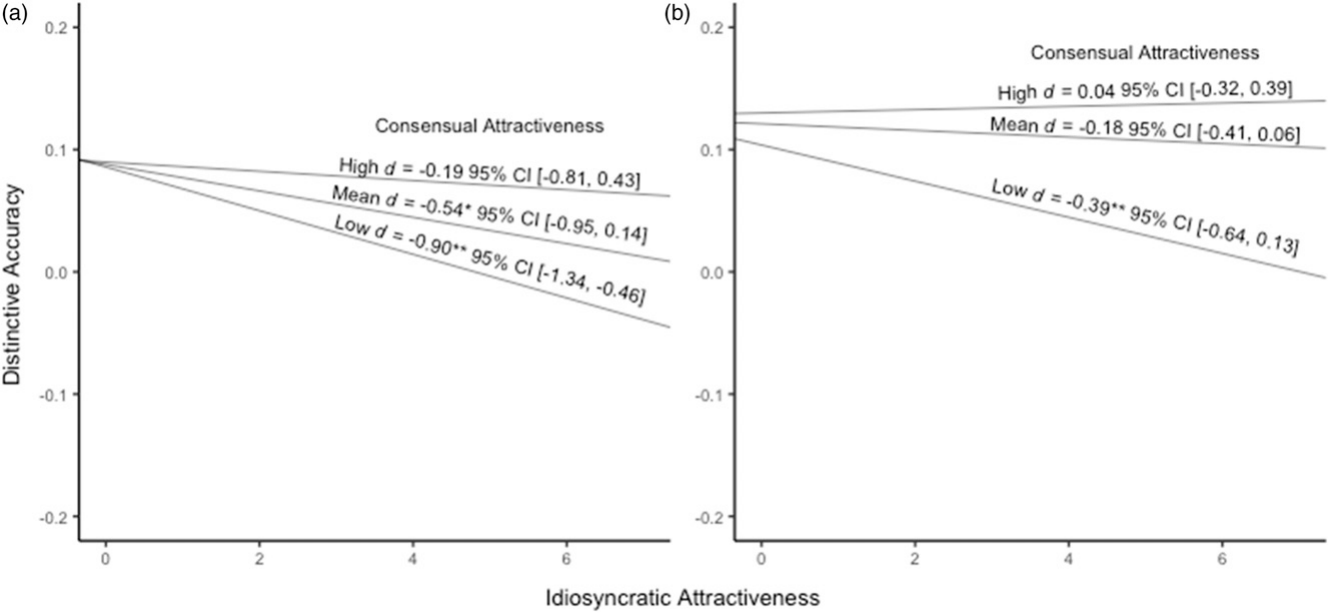
Interaction between targets’ consensual attractiveness and idiosyncratic perceptions of attractiveness on distinctive accuracy. *Note.* Panel A illustrates simple slopes for the association between idiosyncratic attractiveness and distinctive accuracy from the Direct Replication Model at different levels of consensual attractiveness. Panel B illustrates the simple slopes for the association between idiosyncratic attractiveness and distinctive accuracy from the Fully Extended Model. * p < .05, ** *p* < .01.

As in the original study (*b = .*04; Lorenzo et al., 2010), targets’ consensual attractiveness significantly moderated the link between perceiver’s idiosyncratic ratings of target attractiveness and distinctive accuracy, *b* ***=*** 0.01, *t* = 1.97, *p* = .049 (see Figure 2, panel A). Of note, the nature of the simple slopes differed from the original study. Specifically, for targets higher in consensual attractiveness, idiosyncratic attractiveness was not significantly related to distinctive accuracy, *b* ***=*** −0.004, *d* = −0.19, *95% CI* [−0.81, 0.43]*, t* = −0.60, *p* = .54, whereas in the original study it was significantly positive (*b* = 0.08; Lorenzo et al., 2010). For targets lower in consensual attractiveness, greater idiosyncratic attractiveness was related to significantly lower distinctive accuracy, *b* ***=*** −0.02, *d* = −0.90, *95% CI* [−1.34, −0.46]*, t* = −3.99, *p* < .001 (*b* = −0.01; Lorenzo et al., 2010). Therefore, while the original study suggested that finding a target especially attractive may enhance distinctive accuracy for targets generally viewed as more attractive, the present findings suggest that perceiving a target as especially attractive may hinder distinctive accuracy for targets generally viewed as less attractive.

#### Fully extended model

In this second set of analyses, using the extended social accuracy model, we first examined whether people’s impressions were simultaneously characterized by positivity, normative accuracy, and distinctive accuracy. Results revealed that, on average, perceivers viewed targets in line with the socially desirable personality profile, *b* ***=*** 0.73, *t* = 50.38, *p* < .001, the normative personality profile, *b* ***=*** 0.35, *t* = 20.11, *p* < .001, and targets’ distinctive personality profiles, *b* ***=*** 0.12, *t* = 12.11, *p* < .001. As such, following the brief interactions, perceivers viewed targets’ personality profiles positively, and with normative and distinctive accuracy.

##### Attractiveness and positivity of impressions

Were targets’ consensual attractiveness and perceivers’ idiosyncratic ratings of targets’ attractiveness related to the positivity of perceivers’ impressions? Consensual attractiveness was not significantly related to positivity, *b* ***=*** 0.02, *d* = 0.10, *95% CI* [-0.03, 0.23]*, t* = 1.48, *p* = .140. Contrary to the “what is beautiful is good” stereotype, more consensually attractive targets were not viewed more positively, when beautiful was defined more objectively, by consensus. However, consistent with the original findings and those reported in the Direct Replication Model, perceivers’ ratings of targets’ idiosyncratic attractiveness was significantly associated with more positive impressions, *b* ***=*** 0.07, *d* = 0.47, *95% CI* [0.37, 0.56]*, t* = 9.81, *p* < .001 (see Figure 1, Panel B). It seems that finding a target especially attractive reliably predicts viewing that target’s personality more positively, and these results are consistent with the “what is beautiful is good” stereotype, when beauty is in the eye of the beholder. Finally, consistent with the original findings, consensual attractiveness did not significantly moderate the link between idiosyncratic attractiveness and positivity of impressions, *b* ***=*** 0.01, *t* = 1.19, *p* = .233.

##### Attractiveness and normative accuracy

We observed a significant negative link between targets’ consensual attractiveness and normative accuracy, *b* ***=*** −0.08, *d* = −0.44, *95% CI* [−0.64, −0.24]*, t* = −4.29, *p* < .001, suggesting that targets who were generally viewed as more attractive were viewed with less normative accuracy, once positivity was removed. Idiosyncratic attractiveness was not significantly related to normative accuracy, *b* ***=*** −0.01, *d* = −0.07, *95% CI* [−0.22, 0.08]*, t* = −0.87, *p* = .387, and this link was not significantly moderated by consensual attractiveness, *b* ***=*** *−*0.01, *t* = −0.80, *p* = .424. As such, finding a target especially attractive was not necessarily related to viewing their normative personality profiles more accurately.

##### Attractiveness and distinctive accuracy

Consistent with the findings from the Direct Replication Model, targets’ consensual attractiveness was not significantly related to distinctive accuracy, *b* ***=*** 0.02, *d* = 0.36, *95% CI* [−0.13, 0.85]*, t* = 1.42, *p* = .156. Further, the negative association between idiosyncratic attractiveness and distinctive accuracy observed in the Direct Replication Model was no longer statistically significant, *b* ***=*** −0.004, *d* = −0.13, *95% CI* [−0.32, 0.07]*, t* = −1.29, *p* = .198. As such, perceivers did not more accurately perceive the unique personalities of targets who were generally seen as more attractive, or those whom they viewed as especially attractive.

However, consistent with the original study and the Direct Replication Model, consensual attractiveness again significantly moderated the link between idiosyncratic attractiveness and distinctive accuracy, *b* ***=*** 0.01, *t* = 2.01, *p* = .045 (see Figure 2, panel B). In line with the Direct Replication Model above, for targets higher in consensual attractiveness, idiosyncratic attractiveness was not significantly related to greater distinctive accuracy, *b* ***=*** 0.001, *d* = 0.04, *95% CI* [−0.32, 0.39]*, t* = 0.19, *p* = .848, whereas for targets lower in consensual attractiveness, greater idiosyncratic attractiveness was associated with significantly lower distinctive accuracy, *b* ***=*** −0.01, *d* = −0.39, *95% CI* [−0.64, −0.13]*, t* = −2.96, *p* = .003. This indicates that viewing a target as especially attractive could impair distinctive accuracy for targets who were generally viewed as less attractive. See Table 2 for a summary of all associations between attractiveness and accuracy and positivity.

**Table 2.** Summary of results from the direct replication model and the fully extended model.

Model	Perceptual outcome	Predictors							
		Consensual attractiveness			Idiosyncratic attractiveness			Interaction Between Idiosyncratic Attractiveness and Consensual Attractiveness	
		*b*	*d*	*t*	*b*	*d*	*t*	*b*	*t*
Direct replication (Model 1)	Normative accuracy	0.05*	0.22	2.51	0.11**	0.64	13.26	0.01	1.03
	Distinctive accuracy	0.002	0.08	0.17	*−*0.01**	−0.63	−3.00	0.01*	1.97
Fully extended (Model 4)	Positivity of impressions	0.02	0.10	1.48	0.07**	0.47	9.81	0.01	1.19
	Normative accuracy	*−*0.08**	−0.44	−4.29	*−*0.01	−0.07	−0.87	*−*0.01	−0.80
	Distinctive accuracy	0.02	0.36	1.42	*−*0.004	−0.13	−1.29	0.01*	2.01

*Note.* Model 1 estimated normative and distinctive accuracy with self-reports as the accuracy criterion. Model 4 estimated positivity of impressions in addition to normative and distinctive accuracy and used the average of self- and close-other reports as the accuracy criterion.

**p < .01, *p < .05, †*p* < .10.

#### The role of attention

Why would viewing a target as especially attractive result in lower distinctive accuracy for less consensually attractive targets? Perhaps, greater idiosyncratic attractiveness promotes greater attention, but rather than aiding accuracy, this may hinder accuracy because targets lower in consensual attractiveness may not provide highly relevant cues. To test this possibility, we first examined whether idiosyncratic attractiveness predicted greater idiosyncratic attention, controlling for consensual attractiveness and consensual attention. Greater idiosyncratic attractiveness was associated with significantly greater idiosyncratic attention, *b* ***=*** 0.38, *d* = 0.86, *95% CI* [0.76, 0.95]*, t* = 17.50, *p* < .001, suggesting that, perceivers paid more attention to targets who they, idiosyncratically, believed were more attractive.

In turn, does paying greater idiosyncratic attention to consensually less attractive targets relate to lower distinctive accuracy? We examined whether idiosyncratic attention and consensual attractiveness interacted to predict distinctive accuracy, when controlling for the interaction between consensual attractiveness and consensual attention. The interaction between idiosyncratic attention and consensual attractiveness significantly predicted the distinctive accuracy slope, *b* ***=*** 0.02, *t* = 2.60, *p* = .009. The pattern of simple slopes paralleled the earlier findings with idiosyncratic attractiveness, consensual attractiveness, and distinctive accuracy. Specifically, for targets higher in consensual attractiveness, greater idiosyncratic attention was not significantly related to greater distinctive accuracy, *b* ***=*** 0.01, *d* = 0.23, *95% CI* [−0.09, 0.54], *t* = 1.42, *p* = .154. For targets lower in consensual attractiveness, greater idiosyncratic attention was significantly related to lower distinctive accuracy, *b* ***=*** −0.01, *d* = −0.29, *95% CI* [−0.55, −0.03], *t* = −2.14, *p* = .032. Thus, it appears that paying more idiosyncratic attention to targets who were generally viewed as less attractive was related to viewing those targets’ unique personalities less accurately.

Does attention help to explain why targets’ consensual attractiveness moderated the link between idiosyncratic attractiveness and distinctive accuracy? To test this, we added the original interaction between idiosyncratic and consensual attractiveness to the model to see whether perceivers’ idiosyncratic attention accounted for this link. Results revealed that consensual attractiveness continued to significantly moderate the link between perceivers’ idiosyncratic attention and distinctive accuracy, *b* ***=*** 0.01, *t* = 2.04, *p* = .043. Importantly, when controlling for idiosyncratic attention, the interaction between consensual attractiveness and idiosyncratic attractiveness did not significantly predict the distinctive accuracy slope, *b* ***=*** 0.01, *t* = 1.29, *p* = .195. Thus, these results are consistent with the possibility that idiosyncratic attention may account for the interaction between consensual and idiosyncratic attractiveness to predict distinctive accuracy. In other words, perceivers are less accurate when they find a consensually less attractive target more attractive because they pay more attention to that target. However, we did not formally test for causal mediation due to the cross-sectional nature of these data and the complexity of these analytical models.

### Discussion

Is what is beautiful good and more accurately understood? The present study attempted a direct replication and extension of Lorenzo and colleagues’ (2010) findings by (1) using a more reliable accuracy criterion, (2) parsing out positivity from normative accuracy, and (3) exploring the role of attention in these associations. Overall, the current findings partially replicated Lorenzo et al. (2010) but also deviated in several important ways. In general, perceivers accurately recognized targets’ normative and distinctive personality profiles following the brief getting-acquainted interaction. Was attractiveness related to normative and distinctive accuracy levels? In Lorenzo et al. (2010), perceivers viewed targets who were generally viewed as more attractive and those whom they found especially attractive with greater normative accuracy, which was interpreted as a positivity effect, as the normative profile strongly overlaps with social desirability (Rogers & Biesanz, 2015; Wood & Furr, 2016). This finding was replicated in the Direct Replication Model (across all accuracy criteria, see SOM for Models 2 and 5), supporting the “what is beautiful is good” effect. In the extended models, where we parsed out normativity and positivity, seeing a target as especially attractive was associated with viewing that target more positively (across all accuracy criteria, see SOM for Models 3 and 6), closely replicating the interpretation from Lorenzo et al. (2010). However, we did not find evidence that targets who were generally viewed as more attractive were seen more positively. Therefore, our findings support that the “what is beautiful is good” effect may lie more in the eye of the beholder (Douglas & Shepard, 1998; Hönekopp, 2006). Further, normative accuracy was significantly and negatively associated with consensual attractiveness and was not significantly associated with idiosyncratic attractiveness. Thus, once positivity was removed, highly consensually attractive people’s normative personalities were less accurately perceived by others. This finding is consistent with other work parsing out normative accuracy from positivity, demonstrating that greater liking is associated with lower normative accuracy (Wessels et al., 2020).

In terms of distinctive accuracy, we did not find consistent evidence for the original positive association between targets’ consensual attractiveness and distinctive accuracy. Consensual attractiveness was only significantly related to distinctive accuracy when considering close-other reports as the accuracy criterion (see SOM for Models 5 and 6), but this association was not significant in our pre-registered analyses using target self-reports or the average between targets’ and close-other reports as the accuracy criterion. Thus, given our larger sample size and high statistical power, this suggests that more consensually attractive targets’ unique personalities were not necessarily perceived more accurately by others. Further, idiosyncratic attractiveness was related to lower distinctive accuracy in both models, though not significantly in the Fully Extended Model (and in Model 2, see SOM). Distinctive accuracy was positively related to idiosyncratic attractiveness when considering close-other reports as the accuracy criterion (see Model 5 in SOM), but this effect went away when we additionally controlled for positivity in the model (see Model 6 in SOM). Overall, it appears that people do not more accurately view the personalities of targets whom they perceive as being especially attractive, or of targets who are generally considered as attractive. These null associations could be explained by the multiplicative nature of the RAM, suggesting any one stage (e.g., relevance or detection) alone may not be sufficient for achieving accuracy of impressions. For example, consensually attractive targets may be better targets and convey more relevant cues than less consensually attractive targets, but perceivers must also pick up on these cues.

Consistent with this idea and in line with Lorenzo et al. (2010), we found that consensual attractiveness reliably moderated the link between idiosyncratic attractiveness and distinctive accuracy across all models, although this interaction did not reach significance in Model 3 (see SOM for Models 2, 3, 5, and 6). However, while the direction of this interaction was consistent with the original findings, the simple slopes revealed a different pattern. In the original study, idiosyncratic attractiveness was significantly positively associated with distinctive accuracy at higher levels of consensual attractiveness. That is, finding a target especially attractive was associated with viewing that target’s unique personality more accurately if they were also generally viewed as more attractive. Although we found this pattern when considering close-other reports as accuracy criterion (see SOM for Models 5 and 6), this analysis was not pre-registered and the more reliable finding from the present study is that finding a target especially attractive was associated with viewing that target’s unique personality *less* accurately if they were also generally viewed as *less* attractive. In other words, if Tyler was rated as being low in attractiveness by consensus, then the more Paige rated Tyler’s attractiveness as being high, the less accurate she was in perceiving his unique personality profile. Thus, the original study might have captured the higher end of this process, and not fully captured the lower end, perhaps by chance including primarily more attractive targets. In contrast, the present study, which was much larger, may have captured the more typical distribution of attractiveness. Alternatively, perhaps the samples were similar in their attractiveness levels and range, but other sample differences may have contributed to differential patterns of results, including, but not limited to, gender composition (84.8% females in the present study vs. 76.6% in the original study), east coast versus west coast perspectives, and other demographic differences, such as ethnic differences, between universities and geographic regions. Examining sources of the differences would be an interesting avenue for future research. Taken together, the finding that the link between idiosyncratic attractiveness and accuracy depends upon consensual attractiveness appears to be replicable across studies. However, we note that although this analysis was pre-registered, the result was not, and the interaction was not highly statistically significant (and not significant in Model 3) suggesting this may not be a very large effect. Moreover, given the variation in the simple effects across studies, those associations may not be as replicable or stable.

Nevertheless, despite the different pattern of simple slopes across studies, the results may have the same underlying explanation. Perceivers’ idiosyncratic attractiveness may elicit greater attention towards targets (Langlois et al., 2000; Maner et al., 2003). In turn, attention may enhance distinctive accuracy, accuracy about a target’s unique personality, for targets high in consensual attractiveness, as observed in the original study, who might provide more relevant cues due to greater social confidence (Eagly et al., 1991; Feingold, 1992; Reis et al., 1980). In contrast, paying greater attention to less consensually attractive targets may reduce distinctive accuracy, as observed in the present study. One reason this could be is that less attractive targets may be less socially confident (Eagly et al., 1991; Feingold, 1992; Reis et al., 1980), which could impair their ability to accurately express their personalities, assuming they are motivated to do so. As such, these targets may provide irrelevant or misleading signals, which, as per RAM, could reduce accuracy if perceivers pay more attention to them. Targets who are more socially confident may be better at expressing their personalities quickly and efficiently, whereas those who are less socially confident may experience greater difficulty to do so. This is parallel to previous findings showing that paying greater attention to targets higher in well-being, who provide more relevant cues, is associated with greater accuracy, but paying greater attention to targets lower in well-being does not (Human et al., 2014).

Given that distinctive accuracy at first impressions in platonic contexts tends to be related to greater liking, both in the short- and longer-term (Human et al., 2013, 2020), it appears the less attractive people are at a greater disadvantage than more attractive people during first impressions. That is, even if a perceiver does view a target who is consensually less attractive as more attractive, this may actually result in the perceiver seeing their personality less accurately, which could reduce liking.

Lastly, our analyses with attention suggest one possible mechanism that may help explain the associations between attractiveness and accuracy. Specifically, perceivers paid greater attention to those they idiosyncratically found more attractive, which is consistent with previous work demonstrating that more attractive people elicit greater attention from perceivers (Langlois et al., 2000; Maner et al., 2003). However, for targets who were lower in attractiveness, as per consensus, perceivers who reported viewing them as especially attractive viewed their distinctive profiles less accurately. In other words, if Tyler was rated as being less attractive by the group, then the more Paige paid attention to Tyler, the less accurate she is in perceiving his unique personality. Notably, perceivers’ idiosyncratic attention seemed to account for this association. That is, Paige was less accurate about Tyler because she paid more attention to him, suggesting that Tyler, and other targets that perceivers generally viewed as less attractive, may provide irrelevant or misleading cues, thereby hindering the accuracy of impressions.

#### Limitations and future directions

Consistent with Lorenzo et al. (2010), the present study employed the profile approach to examine the associations between attractiveness and normative and distinctive accuracy. Specifically, in contrast to the trait-wise approach, the profile approach provides a more holistic index of the perceiver’s normative and distinctive accuracy about the target’s personality as a whole. Others have also employed the profile approach to make inferences about specific trait-domains (e.g., Liu & Ilmarinen, 2020), which could be an interesting extension of the present work. In the present research, we did not have trait-specific hypotheses, and therefore examined accuracy for all items simultaneously to obtain a more reliable indicator of accuracy and reduce the number of individual analyses. Nevertheless, future research may wish to examine accuracy for specific personality traits and their associations with attractiveness.

It is also important to acknowledge the limitations associated with the cross-sectional nature of the present study. For example, we are unable to establish the directionality of these associations. Nevertheless, much of the previous work (Langlois et al., 2000), including experimental research (Eagly et al., 1991; Feingold, 1992), has argued that attractiveness *influences* the positivity of impressions. Relatedly, manipulations of liking versus disliking have also been found to influence the accuracy, normativity, and positivity of first impressions (Zimmermann et al., 2018). Thus, there is reason to believe that attractiveness may influence how people judge others’ personalities. That said, there is also reason to believe that these associations could be bidirectional as personality judgments could also influence perceptions of attractiveness (e.g., Faust et al., 2018; Gross & Crofton, 1977; Kniffin & Wilson, 2004). Additionally, given the longitudinal evidence suggesting that accuracy could foster liking (Human et al., 2020), it is also plausible that the accuracy of impressions could influence idiosyncratic attractiveness ratings. For example, for consensually less attractive targets, forming less accurate impressions might actually promote idiosyncratic attractiveness ratings. That is, for targets who may possess less appealing qualities, perceivers may be more likely to report greater idiosyncratic attractiveness if the perceivers are less accurate at perceiving the targets’ less appealing qualities (Kerr et al., 2020). Furthermore, in the present study, we did not formally test for causal mediation to explore the role of attention. Nevertheless, these findings were consistent with mediation, and therefore provide preliminary evidence for a possible mediating role of attention in the links between attractiveness and accuracy at first impressions. Additional research is needed to better understand the directionality of the results reported here.

The present study is also unable to determine what motivations, if any, underlie how accurately targets express themselves to others. Self-verification theory (Swann, 1983) would suggest that most people wish to be seen in line with their self-views and may actively attempt to elicit such impressions from others, which may require some social confidence. However, it is also possible that this is a more unintentional process, or that some targets may wish to be seen inaccurately. That is, were targets who were less accurately perceived unable to accurately express their personalities, despite their best efforts, or did they purposely provide misleading information? And are such motivations and attempts conscious or unconscious? Determining the mechanisms through which attractiveness might influence the quality and quantity of cues and in turn influence accuracy is an important next step for future research.

Another caveat of the present study is that attention was indexed by participants’ self-reported level of engagement and interest in their interaction partner. The phrasing of this item may contain a positive connotation for some participants, which may have been confounded with their general level of liking of the target. As such, it may be interesting for future research to employ more objective ways to assess attention during first impressions, such as using eye-tracking as an index of attention (see Capozzi et al., 2019).

Additionally, 84.8% of the present sample was composed of women (similar to the 76.6% in the original study), which resulted in mostly same-gender interactions (75.6%). Given this large imbalance, the present data do not lend itself to testing whether the type of gender configuration moderated any of the associations between attractiveness, accuracy, and attention. Thus, it may be interesting for future research to examine the role of same-gender versus opposite-gender interactions within platonic settings. People may have different self-presentational concerns and motivations when engaging in interactions with targets who are of one’s romantically preferred gender (Sprecher & Regan, 2002). Indeed, accuracy in romantic first impressions contexts, such as during speed-dating, has been differentially related to interpersonal outcomes (Kerr et al., 2020) as compared to platonic contexts (Human et al., 2020). Specifically, in platonic contexts, greater distinctive accuracy of first impressions was related to greater liking of new acquaintances (Human et al., 2020), whereas the opposite pattern was observed in a romantic context, such that greater distinctive accuracy was related to lower romantic interest (Kerr et al., 2020). As such, opposite-sex interactions, to the extent they provide an opportunity for romantic relationship initiation, could be an important moderator to consider.

Further, we did not have an assessment of the quality of behavioral cues to determine whether it was indeed less relevant cues that led to this negative link between attention and accuracy for less consensually attractive targets. Having a direct, behavioral measure of cue relevance is needed to establish whether this underlies the association between targets’ consensual physical attractiveness and their expressive accuracy during first impressions. Additional research is also needed to more directly examine whether less attractive targets provide lower quality cues in general or particularly when interacting with perceivers who find them attractive, an interesting avenue for future research.

## Conclusion

In sum, the present results support the idea that attractiveness relates to both the accuracy and positivity of personality impressions. We found support for the idea that “what is beautiful to the beholder is good,” such that when perceivers judged targets to be more attractive, they also viewed their personalities more positively. However, we did not find consistent support that targets who were generally viewed as more attractive were viewed more positively, as was observed in the original study by Lorenzo et al. (2010). In terms of accuracy, we did not replicate the original study’s key finding that targets who were generally viewed as more attractive were seen more accurately. However, consistent with the original study, we found an interaction between consensual and idiosyncratic attractiveness predicting accuracy. That is, the association between how idiosyncratically attractive a perceiver found a target and how accurately the perceiver viewed that target’s personality depended upon the target’s consensual attractiveness level. However, the nature of the interaction did differ from the original study. Specifically, it appears that what is subjectively beautiful is good, as indexed by perceivers’ idiosyncratic impressions, but, according to the present study, may be less accurately understood if beauty is objectively low, as defined by consensus. This could be because perceivers pay greater attention to targets whom they deem more attractive, which could hinder accuracy if those targets are harder to read.

## Supplemental Material

Supplemental Material - Is what is beautiful good and still more accurately understood? **A** replication and extension of Lorenzo et al. (2010) Supplemental Material for Is what is beautiful good and still more accurately understood? A replication and extension of Lorenzo et al. (2010) by Hasagani Tissera, John E Lydon and Lauren J Human in European Journal of Personality

## Notes

1. Consistent with previous work (Wood & Furr, 2016), the normative and the socially desirable (i.e., positive) personality profiles were highly correlated (*r* = .82). Despite the high correlation, it is possible to disentangle these two components, as seen in previous work (e.g., Rogers & Biesanz, 2015; Wessels et al., 2020; Zimmermann et al., 2018). Further, to ensure that there were no problems with multicollinearity, we examined the VIF coefficients. In the extended model, estimating both normativity and positivity simultaneously, the VIF coefficients ranged between 1 and 1.5, suggesting that multicollinearity is not a cause for concern.

2. Although it was not pre-registered, we screened the data for inattentive responding. To do so, we employed the “careless” package for R (Johnson, 2005) in which we identified long strings of consecutive responses with the same response option. To decide on a cutoff point, we employed a scree-like test where we visually examined for a dip in frequency of consecutive responses. For partner-, self- and close-other ratings, the cutoff was determined to be 7 consecutive responses. This involved excluding 2.5% of the partner-ratings, 2.2% of the self-reported data and 1.5% of the close-other data. The *R* code for identifying careless responses is available online at https://osf.io/64txm/. When we re-ran the analyses after excluding these cases, we obtained a pattern of results consistent with those reported here.

3. For completeness, we also explored whether the pattern of results was consistent when using the close-other reports as the accuracy criterion. The findings were largely consistent with those reported here. We report on these results in the SOM. These analyses were not pre-registered
