# Supplementary material for: Is what is beautiful good and still more accurately understood? A replication and extension of Lorenzo et al. (2010)
Source: Eur J Pers. 2022 May 17;37(4):468–84. doi: 10.1177/08902070221099688 (PMC13021046; doi:10.1177/08902070221099688)
Supplement: Supplemental Material - Is what is beautiful good and still more accurately understood? A replication and extension of Lorenzo et al. (2010) [file sj-pdf-1-erp-10.1177_08902070221099688.pdf]

Supplementary Online Materials

Self-Other Correlations for Personality Items

Item	Correlations between Target's Self-Report and Perceiver Ratings (*r*)
Is full of energy	*.18*
Is intelligent	*.03*
Generates a lot of enthusiasm	*.20*
Remains calm in tense situations	*.07*
Tends to be quiet	*.27*
Makes plans and follows through with them	*.02*
Has an assertive personality	*.12*
Is sometimes shy, inhibited	*.19*
Is outgoing, sociable	*.22*
Tends to find fault with others	*.01*
Does a thorough job	*.001*
Is depressed, blue	*.03*
Is original, comes up with new ideas	*.05*
Is helpful and unselfish with others	*.04*
Can be somewhat careless	*.07*
Is relaxed, handles stress well	*.06*
Receives very good grades	*.07*
Starts quarrels with others	*.07*
Is a reliable worker	*.02*
Can be tense	*-.001*
Is reserved	*.19*
Is ingenious, a deep thinker	*.07*
Has a forgiving nature	*-.004*
Is bright	*.06*

Additional Preregistered Analyses: Results for Models 2 and 3

As preregistered, we report results of two other models (Model 2 and Model 3) that build on the original findings from Lorenzo, Biesanz & Human (2010), not discussed in detail in the primary manuscript. Model 2 was an extension of the original model in Lorenzo et al. (2010) and Model 1, the Direct Replication Model, presented in the manuscript, with the use of a more reliable accuracy criterion: the average of self and close other reports of targets' personalities. *Therefore, Model 2 followed an identical analytical approach as Model 1 (see manuscript), except for using* the average of both self- and close other reports of targets' personalities *as the accuracy criterion.* Model 3 was an extension of the original model that parsed out normative accuracy from positivity (like Model 4, the Fully Extended Model, in the manuscript) but using the same accuracy criterion as the original study: target self-reports. Accordingly, the analytical approach for Model 3 was identical to Model 4 (see manuscript), with the exception of using targets' self-reported personalities as the accuracy criterion. Table S1 and Figure S1 summarize the results from Models 2 and 3. Overall, results from Model 2 were largely consistent with those from Model 1, and the results from Model 3 were highly consistent with those from Model 4. R code and data for re-creating these analyses can be found at https://osf.io/64txm/?view_only=77b06b4b7fa242869b35a541543d1665.

Table S1

Summary of Results From Model 2 and 3

Model	Perceptual Outcome	Predictors							
		Consensual Attractiveness			Idiosyncratic Attractiveness			Interaction Between Idiosyncratic Attractiveness and Consensual Attractiveness	
		b	*d* *[95% CI]*	*t*	*b*	*d* *[95% CI]*	*t*	*b*	*t*
2	Normative Accuracy	0.05**	0.23 [0.11, 0.35]	3.80	0.10**	0.60 [0.52, 0.68]	13.99	0.02*	2.14
	Distinctive Accuracy	0.03*	0.73 [0.01, 1.45]	1.99	-0.01	-0.21 [-0.52, 0.11]	-1.27	0.02*	2.33
3	Positivity of Impressions	0.02	0.08 [-0.08, 0.26]	1.02	0.08**	0.55 [0.45, 0.66]	10.09	0.002	0.26
	Normative Accuracy	-0.08**	-0.47 [-0.69, -0.24]	-4.03	0.001	0.01 [-0.15, 0.17]	0.12	-0.02	-1.31
	Distinctive Accuracy	-0.01	-0.14 [-0.65,0.37]	-0.55	-0.01**	-0.36 [-0.60, -0.11]	-2.94	0.01	1.61

Note. Model 2 was an extension of Model 1, with the use of the average of self- and close other reports of targets' personalities as the accuracy criterion. Model 3 parsed out normative accuracy from positivity (like Model 4) but used targets' self-reports as the accuracy criterion.

**p < .01, *p < .05, †p < .10

Figure S1

Interactions Between Targets' Consensual Attractiveness and Perceptions of Idiosyncratic Attractiveness Predicting Normative Accuracy and Distinctive Accuracy in Model 2

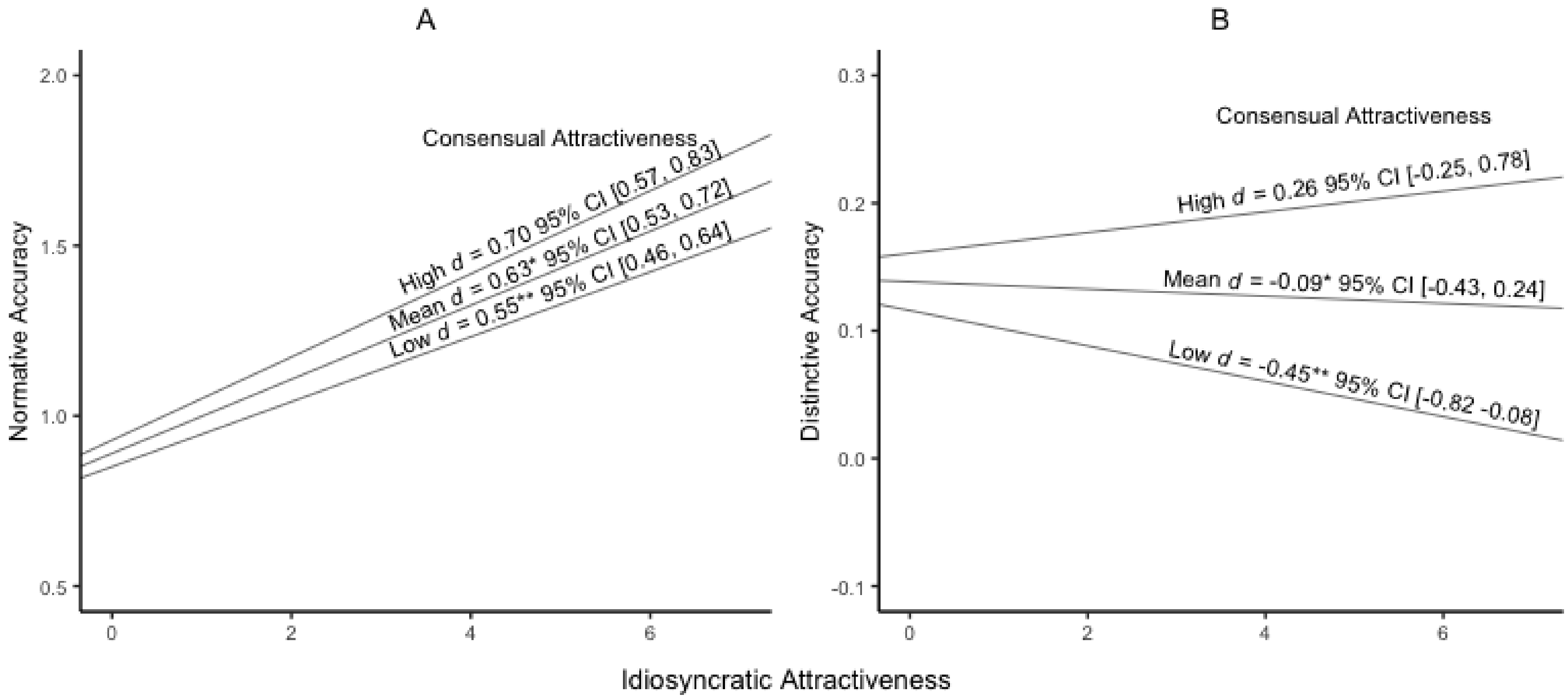

Note. Panel A illustrates the association between idiosyncratic attractiveness and normative accuracy at different levels of consensual attractiveness. Panel B illustrates the association between idiosyncratic attractiveness and distinctive accuracy at different levels of consensual attractiveness. ** $p < .01$

Additional Preregistered Analyses: The Role of Attention.

*A*s stated in our preregistration, we explored whether perceivers' self-reported attention accounted for any significant associations that emerged in Model 4, the most reliable and conservative model. In this model, 1) consensual attractiveness was related to lower normative accuracy, 2) idiosyncratic attractiveness was related to greater positivity of impressions, and 3) consensual and idiosyncratic attractiveness interacted to predict distinctive accuracy. We reported whether attention contributed to the interaction between consensual and idiosyncratic attractiveness to predict distinctive accuracy in the main manuscript. Here, we report on the possible role of consensual attention in the link between consensual attractiveness and lower normative accuracy, and the role of idiosyncratic attention in the link between idiosyncratic attractiveness greater positivity of impressions.

Analytical approach

First, does consensual attention contribute to the link between targets' consensual attractiveness and normative accuracy? Using multilevel regression analyses, we examined whether targets' consensual attractiveness was related to consensual attention. We also examined whether consensual attention was related to normative accuracy when controlling for consensual attractiveness, and whether the inclusion of consensual attention accounted for variance in the initial association between consensual attractiveness and normative accuracy. Second, does idiosyncratic attention contribute to the link between idiosyncratic attractiveness and the positivity of impressions? We tested whether idiosyncratic attractiveness predicted idiosyncratic attention. We then examined whether idiosyncratic attention was related to positive personality impressions, above and beyond idiosyncratic attractiveness, and whether the link between idiosyncratic attractiveness and positivity of impressions weakened when controlling for

idiosyncratic attention. Of note, given the cross-sectional nature of these data, we are unable to establish the directionality of these associations.

Results and Discussion

First, could consensual attention help explain why consensual attractiveness was related to lower normative accuracy? Consensual attractiveness was related to significantly greater consensual attention, $b = 0.32$, $d = 0.83$, *95% CI* [0.76, 0.90], $t = 24.40$, $p < .001$, indicating that more consensually attractive targets received greater attention from perceivers on average. Is consensual attention in turn related to normative accuracy, above and beyond targets' consensual attractiveness? Indeed, consensual attention was significantly associated with lower normative accuracy, $b = -0.17$, $d = -0.71$, *95% CI* [-0.92, -0.50], $t = -6.72$, $p < .001$, when controlling for consensual attractiveness. Further, consensual attractiveness was no longer significantly related to normative accuracy when consensual attention was in the model, $b = -0.03$, $d = -0.13$, *95% CI* [-0.34, 0.07], $t = -1.27$, $p = .206$. These results are consistent with the idea that consensual attention may help to explain why more consensually attractive targets were seen less in line with the normative personality profile. Specifically, more consensually attractive targets received greater attention, which was related to being viewed as less normative.

Second, could perceivers' idiosyncratic attention help explain the association between idiosyncratic attractiveness and the positivity of impressions? Given that idiosyncratic attractiveness was related to greater idiosyncratic attention (see manuscript), perhaps this idiosyncratic attention in turn relates to more positive personality impressions. Indeed, idiosyncratic attention was significantly related to more positive impressions, $b = 0.09$, $d = 0.57$, *95% CI* [0.48, 0.66], $t = 12.12$, $p < .001$, when controlling for idiosyncratic attractiveness. Moreover, the association between idiosyncratic attractiveness and positivity weakened, yet

remained significant, when controlling for idiosyncratic attention, $b = 0.04$, $d = 0.28$, *95% CI* [0.18, 0.38], $t = 5.60$, $p < .001$. It seems that perceivers paid greater attention to targets whom they deemed more attractive, which may have partially contributed to the link between idiosyncratic attractiveness and the positivity of personality impressions. Perhaps greater attention fosters a sense of familiarity, which has previously been associated with more positive social interactions (Reis et al., 2011). Thus, paying more attention to a person may also enhance how positively a perceiver views their personality.

Additional Exploratory Analyses: Using Close Other Reports of Targets' Personality As Accuracy Criterion

Although it was not pre-registered, we also explored whether we observed a similar pattern of results when considering the close-other reports as the accuracy criterion (Model 5 and Model 6). Model 5 is parallel to the Direct Replication Model (Model 1) discussed in the main manuscript, except we use close other reports as the accuracy criterion instead of targets' self-reports. *Therefore, Model 5 followed an identical analytical approach as Model 1 (see manuscript).* Model 6 parallels Model 4 presented in the main manuscript, which parses out normative accuracy from positivity but uses close other reports as the accuracy criterion. As such, the analytical approach for Model 6 was identical to Model 4 (see manuscript), with the exception of using close other reports of targets' personalities as the accuracy criterion. Table S2 and Figure S2 summarize the results from Models 5 and 6. Overall, results from Model 5 were largely consistent with those from Model 1, and the results from Model 6 were highly consistent with those from Model 4. R code and data for re-creating these analyses can be found at https://osf.io/64txm/?view_only=77b06b4b7fa242869b35a541543d1665.

Table S2
Summary of Results Using Close Other Reports as Accuracy Criterion

Model	Perceptual Outcome	Predictors							
		Consensual Attractiveness			Idiosyncratic Attractiveness			Interaction Between Idiosyncratic Attractiveness and Consensual Attractiveness	
		b	*d* *[95% CI]*	*t*	*b*	*d* *[95% CI]*	*t*	*b*	*t*
5	Normative Accuracy	0.06**	0.27 [0.12, 0.36]	3.54	0.10**	0.56 [0.46, 0.66]	10.84	0.02†	1.86
	Distinctive Accuracy	0.05**	1.15 [0.40, 1.91]	2.99	0.01*	0.37 [0.03, 0.71]	2.14	0.02*	2.16
6	Positivity of Impressions	0.03†	0.14 [-0.01, 0.30]	1.81	0.07**	0.49 [0.38, 0.60]	8.47	0.02	1.62
	Normative Accuracy	-0.08**	-0.43 [-0.71, -0.17]	-3.19	-0.01	-0.07 [-0.27, 0.12]	-0.77	0.004	0.26
	Distinctive Accuracy	0.04*	0.69 [0.17, 1.22]	2.60	-0.001	-0.02 [-0.24, 0.19]	-0.21	0.02**	2.74

Note. Model 5 parallels Model 1 but used close other reports of targets' personalities as the accuracy criterion. Model 6 parallels Model 4, parsing out normative accuracy from positivity but using close other reports as the accuracy criterion.

**p < .01, *p < .05, †p < .10

Figure S2

Interactions Between Targets' Consensual Attractiveness and Perceptions of Idiosyncratic Attractiveness Predicting Distinctive Accuracy in Models 5 and 6

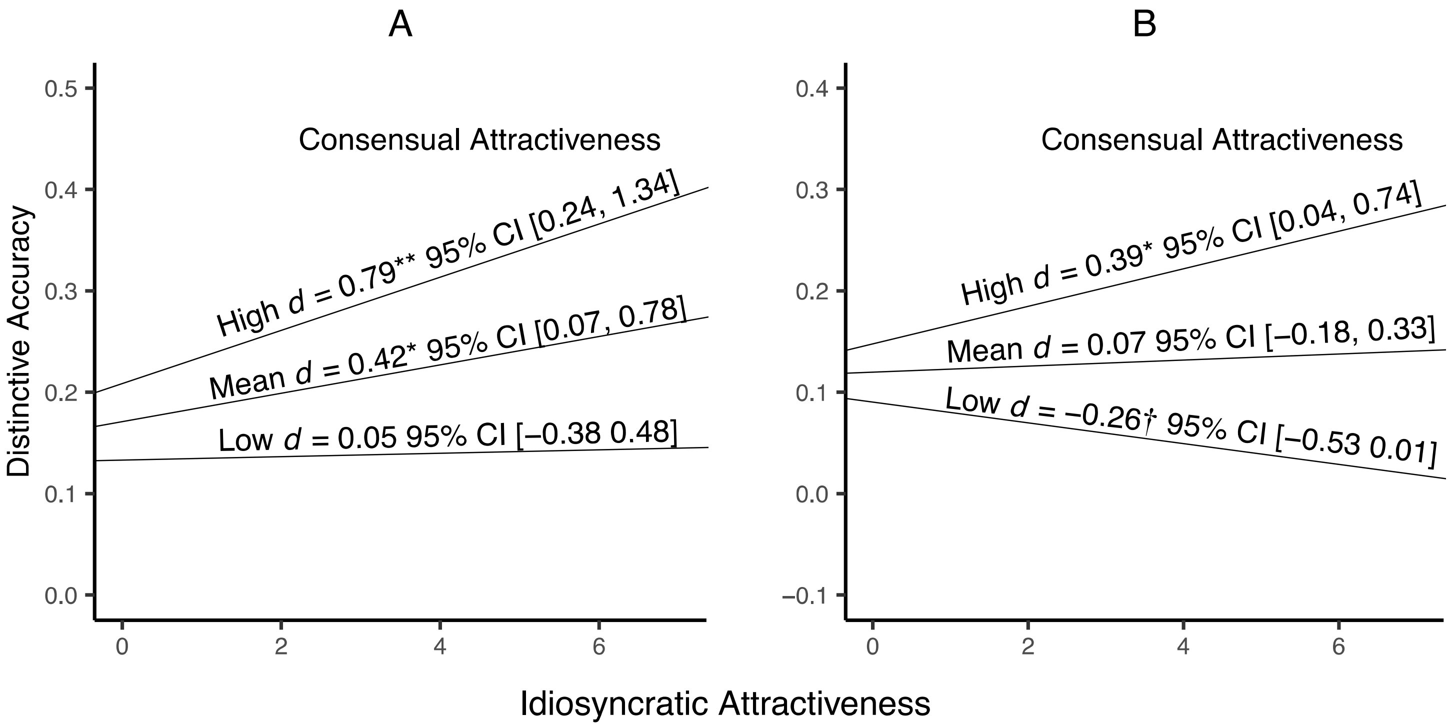

Note. Panel A illustrates the association between idiosyncratic attractiveness and distinctive accuracy at different levels of consensual attractiveness from Model 5, and Panel B illustrates this same association in Model 6. ** $p < .01$, * $p < .05$, † $p < .10$
